# Bandwidth Enhancement of a Dual Band Planar Monopole Antenna Using Meandered Microstrip Feeding

**DOI:** 10.1155/2014/856504

**Published:** 2014-03-03

**Authors:** M. R. Ahsan, M. T. Islam, M. Habib Ullah, N. Misran

**Affiliations:** Department of Electrical, Electronic, and System Engineering, Faculty of Engineering and Built Environment, Universiti Kebangsaan Malaysia (UKM), 43600 Bangi, Selangor, Malaysia

## Abstract

A meandered-microstrip fed circular shaped monopole antenna loaded with vertical slots on a high dielectric material substrate (*ε*_*r*_ = 15) is proposed in this paper. The performance criteria of the proposed antenna have been experimentally verified by fabricating a printed prototype. The experimental results show that the proposed antenna has achieved wider bandwidth with satisfactory gain by introducing meandered-microstrip feeding in assistant of partial ground plane. It is observed that, the −10 dB impedance bandwidth of the proposed antenna at lower band is 44.4% (600 MHz–1 GHz) and at upper band is 28% (2.25 GHz–2.95 GHz). The measured maximum gains of −1.18 dBi and 4.87 dBi with maximum radiation efficiencies have been observed at lower band and upper band, respectively. The antenna configuration and parametric study have been carried out with the help of commercially available computer-aided EM simulator, and a good accordance is perceived in between the simulated and measured results. The analysis of performance criteria and almost consistent radiation pattern make the proposed antenna a suitable candidate for UHF RFID, WiMAX, and WLAN applications.

## 1. Introduction

In the recent years, the area of wireless communications has received an intense boost with innovative research works and advancements by grace of cutting-edge technology. Since low-cost, compact, and reliable wireless communication device has turned into an essential requirement in our daily social life, the request for designing small-size, wideband, multiband, and highly efficient antennas escalated. The wideband and multiband functionalities in antennas are a fundamental requirement nowadays to equip with the communication systems so that it can utilize the space effectively to increase portability and satisfy the standard operating frequency bands [1–4]. The microstrip antennas are being increasingly used in communication systems since they inherently have got some promising preferences other than conventional antennas while considering size, cost, manufacturing process, durability, and conformability [5]. On the other hand, intrinsically, the microstrip patch antennas also suffer from narrow bandwidth which in turn deteriorates the performance while covering up some specific bands. The review process unfolds numerous patch antennas of various sizes and shapes of radiating patch with a number of dissimilar techniques that were proposed for widening the impedance bandwidth. Out of the bandwidth broadening techniques, some can be mentioned here as, for example, introducing separate slit lines [6–8], implementing unusual feeding techniques [9, 10], integrating metallic strip surrounding the radiating patch or some of its part as parasitic element [11, 12], using thick substrate or higher dielectric substrate [13], embedding array of similar geometric pattern-based patch structures named differently as electromagnetic band gap (EBG) [14, 15], and metasurface [16], employing metamaterial [17, 18]. Other techniques rely on using parasitic elements to increase the bandwidth [19–23]. However, not all the above mentioned designs can significantly increase the bandwidth and gain of the antenna. Additionally, the existence of trade-off between the complex, time consuming, cost ineffective antenna designs as well between the antenna characteristics can be perceived by revising the associated literature thoroughly.

Some of the recent literature proposing the meandered-line-fed which is a type of microstrip-line-fed patch antenna has received potential interest among the researchers in antenna design since this technique is less cumbersome in widening the bandwidth of patch antenna [24]. The microstrip-line-fed patch antennas ensure several stunning properties as, for example, less radiation loss, low cross-polarization, easy fabrication and integration, and no need to be used via hole in the existing microstrip technology. Taking the advantage from strip-line-fed, a meandered-line-fed circular type patch antenna loaded with slit of similar width has been designed to cover two important frequency bands, namely, ultrahigh frequency (UHF) band for Radio Frequency Identification (RFID) and lower wireless/Worldwide Interoperability for Microwave Access (WiMAX) band. A considerable amount of interest has been paid to the RFID system in UHF band due to its organisational and commercial use for its tracking and identification capabilities. In general, the UHF RFID system functions at the bands of Europe (865–867 MHz) and/or North America (902–928 MHz) [25, 26]. On the other hand, WiMAX is based upon IEEE 802.16-2004, which is later modified to IEEE Std. 802.16e-2005, and it has different spectrum allocation in different part of the world based on the standard bands 2.3 GHz, 2.5 GHz, and 3.5 GHz [27, 28].

In this paper, a new printed antenna configuration alongside the details of obtaining optimal design structure is proposed with a couple of slits on the radiating surface and meandered-structure fed. To demonstrate and analyze the performance characteristics of the proposed antenna, a physical model has been fabricated with a diameter of 38 mm radiating element, substrate thickness of 2 mm, and fed by meandering-microstrip structure. Through numerous parametric studies, it has been revealed that the appropriate placement of slots on the circular radiator along with the advantages of partial ground [29] and meandered-microstrip structure can facilitate to attain the extended impedance bandwidth and good radiation properties which make it suitable for dual band wireless communication systems like UHF RFID and WiMAX applications.

## 2. Antenna Structure

**Figure 1:**
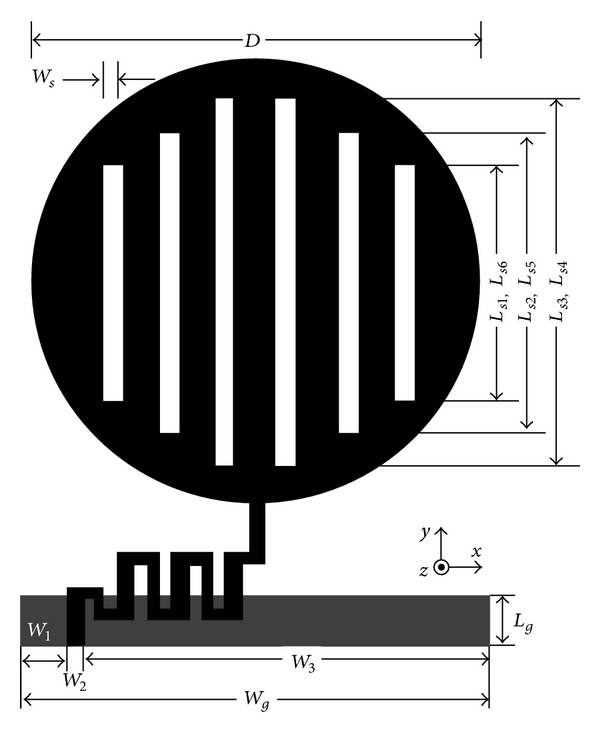
Geometric structure of the meandered-microstrip-fed monopole antenna.

The widely popular, commercially available 3-dimensional full wave high frequency electromagnetic structural simulation (HFSS) tool is used for the design and simulation of the proposed meanderd-microstrip-fed vertical slot loaded circular monopole antenna. Like other typical microstrip patch antennas, the proposed antenna contains an SMA connector in its side, a meandered structure microstrip line to feed the radiating part, a circular type radiating surface introduced with slot line of the same width on top and a rectangular ground plane at the bottom. The meandered-line-fed microstrip monopole antenna structure and its associated detail dimensions are given by Figure 1. The electrical dimension of the radiating element with respect to wavelength in 0.9 GHz is 0.114*λ* × 0.114*λ*. For the case of total antenna structure (same as substrate size), *W* = 41 mm (0.15*λ*), *L* = 51 mm (0.12*λ*), *h* = 2 mm (0.006*λ*). The radiating surface is of circular type where three types of different vertical rectangular slots of similar width (*W*_*s*_ = 1.5 mm) are introduced. The diameter of the circular radiator (*D*) is chosen as 38 mm and the lengths of its narrow rectangular slot lines are 32 mm, 24 mm, and 18 mm for middle slot (*L*_*s*3_, *L*_*s*4_), side slot (*L*_*s*2_, *L*_*s*5_), and outer side slot (*L*_*s*1_, *L*_*s*6_), respectively. Therefore, half of the circular radiating element is symmetrical to the other half along *x*-axis, thus effectively helping enhance the radiation by cancelling the cross-polarization effect. In between the radiating surface and partial ground plane, a 2 mm thick ceramic filled bioplastic substrate of relative permittivity (*ε*_*r*_) 15 is being inserted. The width, *W*_*g*_ (same as substrate), and length, *L*_*g*_, of the ground plane are 41 mm and 5 mm, respectively. The breakdown for the total width *W*_*g*_ is *W*_1_ = 7 mm, *W*_2_ = 1 mm, and *W*_3_ = 33 mm. The radiating element is excited by an optimized meandered microstrip structure which is also coupled to the SMA connector by impedance matching. Since impedance agreement is considered as very much sensitive to the geometry of the feed arrangement, a parametric study has been performed to find out best choice of meandered structure to feed the monopole antenna.

## 3. Parametric Analysis

To reveal most of the proposed antenna's geometric and optimized structure where best impedance matching has been achieved, parametric studies are performed accordingly. For the parametric studies, the numerical simulations have been done by finite element method (FEM) based commercial 3D simulator HFSS, which has a well established reputation in terms of accuracy. The parametric studies consider only the cutting slot inside the circular shaped radiating element and the feeding structure to it. Other monopoles use small hole along the surface to make the antenna broadband instead of multiband [30]. It is already known to the antenna researchers that some of the parameters (e.g., dimension of antenna and feed location) and performances (e.g., gain and radiation efficiency) have effect on the monopole antenna; these are excluded from parametric studies. For better understanding of the impact of the parameter on the antenna performance, only one parameter has been picked for investigation while the other parameters were left as it is.

**Figure 2:**
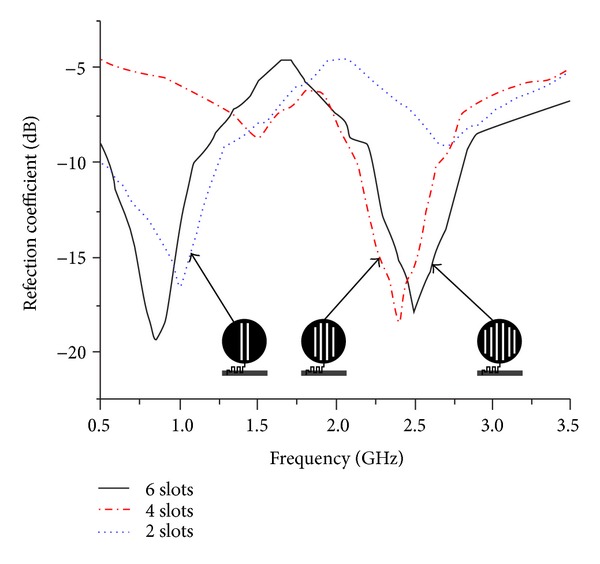
Simulated reflection coefficient for different numbers of slots inside radiating element.

Firstly, the parametric study focuses on cutting slot inside the rounded radiating element and its enumeration while keeping its width similar to *W*_*s*_. The graphical presentation in Figure 2 validates the study and outcome. At the center of the radiating patch, the slots *L*_*s*3_ and *L*_*s*4_ has cut symmetrically, which are placed at the same distance from the central line. In this arrangement, the lower frequency is said to be getting closer to the achieved resonant frequency at impedance bandwidth of less than −10 dB. The symmetrical placement of the two side slots *L*_*s*2_ and *L*_*s*5_ is responsible for the higher frequency band. The outer side slots inside the circular radiator *L*_*s*1_ and *L*_*s*6_ significantly affect the excitation and resonant frequencies that are achieved at 0.9 GHz and 2.5 GHz with less than −10 dB reflection coefficient, −19.4 dB, and −17.9 dB, respectively.

**Figure 3:**
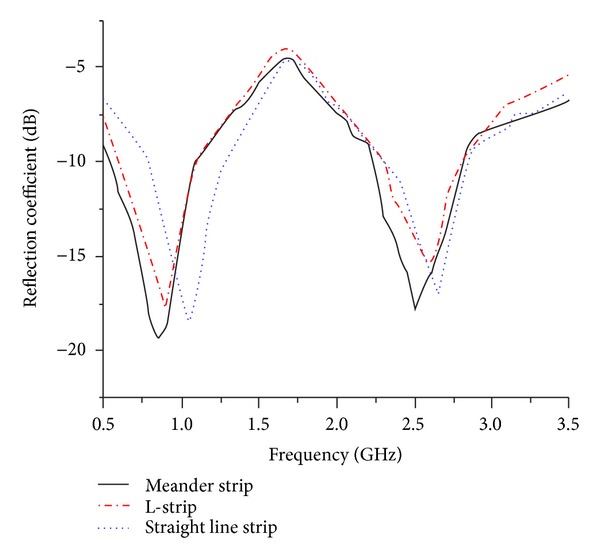
Simulated reflection coefficient for different feeding structures.

Further investigations have been performed on the feeding structure to the slotted circular radiator which is responsible for contributing in the bandwidth widening process through good impedance matching.  Figure 3 illustrates the antenna performance in terms of reflection coefficient for different feeding arrangements. For the case of L-strip and straight microstrip feed, the reflection coefficients at center frequency and bandwidth are close by those of the meander strip feeding. However, under the situation of 50 Ω impedance matching, the meander microstrip feed structure offers the best agreement and exhibits broader bandwidth compared to other feeding methods. Additionally, with the assist of partial ground plane, the meander microstrip structure promotes longer resonant mode by adapting the electric length which plays a vital role in bandwidth enhancement. From the analysis, it has been observed that the meandered type microstrip feeding offers better phase distribution which leads to the reduction of cross-polarization effect.

## 4. Result and Discussion

**Figure 4:**
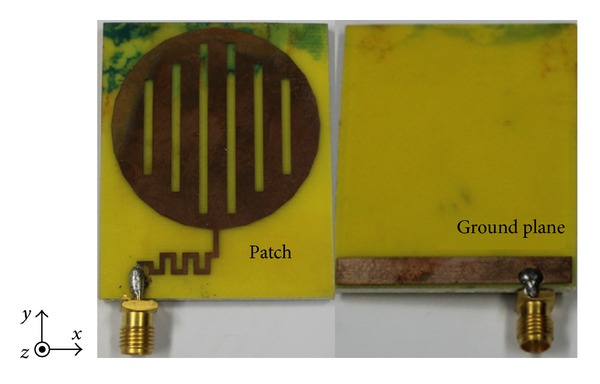
Photograph for the prototype of the proposed antenna.

**Figure 5:**
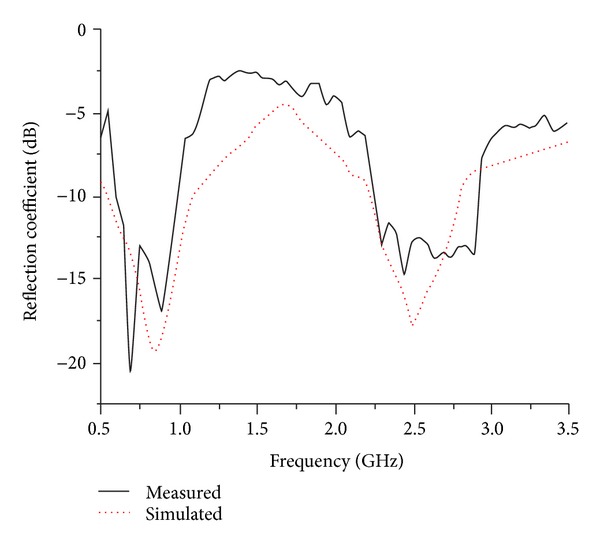
Simulated and measured reflection coefficient of the proposed antenna.

The performance characteristics of the meandered-strip-fed circular type microstrip monopole antenna have been analyzed, studied, and optimized by utilizing the 3D electromagnetic structure solving functionality of ANSYS' FEM based HFSS simulator. The accomplishment of the parametric study gives an optimized geometric structure of the proposed antenna which is realized through in-house PCB LPKF prototyping machine to get a physical test model and is presented in Figure 4. Afterwards, the antenna parameters have been measured with the help of Agilent's Vector Network Analyzer (Agilent E8362C) in a standard sized anechoic measurement chamber. The simulation and measured antenna parameters have been further evaluated and graphically presented by available software package and computer aided tools.  Figure 5 shows the measured and simulated −10 dB reflection coefficients against frequency for the proposed two band antenna. The graphical output undoubtedly presents an excellent agreement between the simulated and measured reflection coefficients. However, a diminutive deviation can be seen in between the simulated and measured results which may occur on behalf of the fabrication tolerance affected by thickness uncertainty and/or existed inconsistency in the substrate material. The measured less than −10 dB impedance bandwidth reflection coefficients range from 600 MHz to 1 GHz (44.4%) and 2.25 GHz to 2.95 GHz (28%), respectively. It is apparent that the attained bandwidth of the proposed antenna can successfully cover the UHF RFID and 2.3/2.5 GHz WiMAX/WLAN bands.

**Figure 6:**
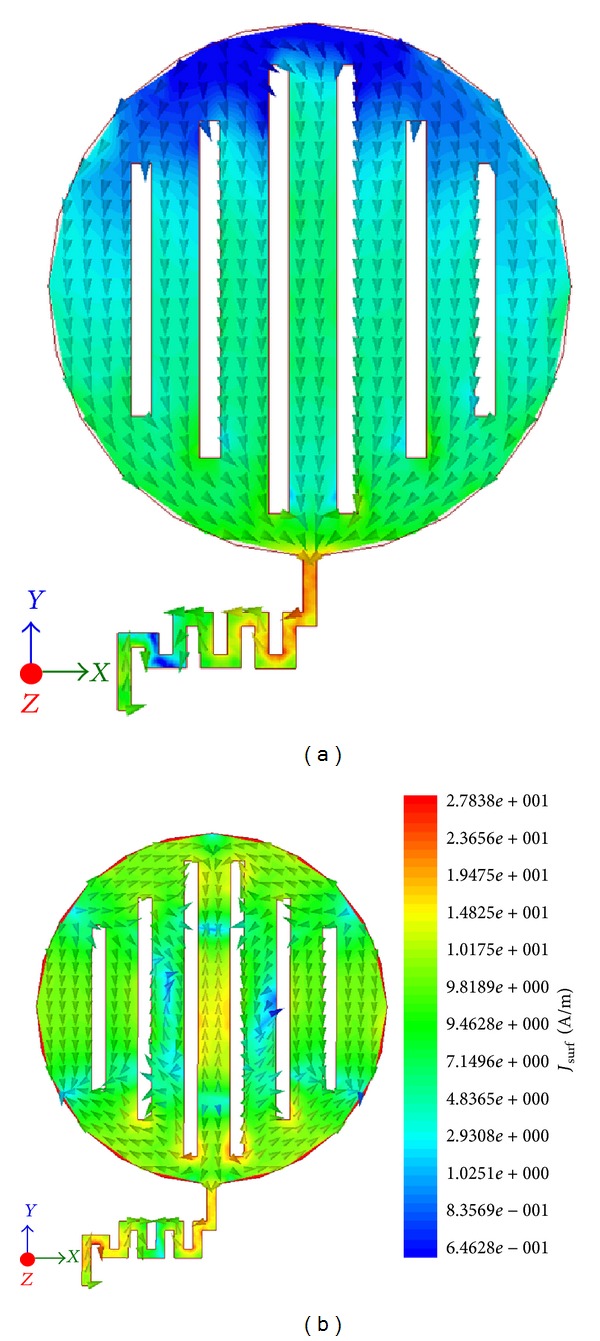
Distribution of surface current at 0.9 GHz and 2.5 GHz.

The simulation software HFSS also provides the distribution of surface currents along the radiating surface and feed line. The proposed antenna is further studied in terms of surface current distribution at two of its resonant frequencies, more specifically at 0.9 GHz and 2.5 GHz, which are furnished through Figure 6. At lower frequency of 0.9 GHz, an increased amount of surface current is seen to flow through the lower part of the radiating element and also nonuniformity of current distribution can be seen along the meandered-stripling-fed structure. The surface current path for this case is less disturbed which leads to generation of almost homogeneous electric and magnetic fields and thus provides less cross-polarization, whereas at high frequency of 2.5 GHz, the distribution of surface current along the radiating surface and meandered-stipline-fed structure is almost uniform except the middle microstrip of the patch which seems to carry slightly more surface currents. Stronger current distributions are also noticeable at the starting and end terminal of the meandered-microstrip-fed structure. Furthermore, in comparison to the lower frequency, the variation of the current phase along the storylines of the radiating element is clearly visible. The resulted effect can be validated through radiation pattern where a slight increase of cross-polarization and little discrepancy in E- and H-field can be realized.

**Figure 7:**
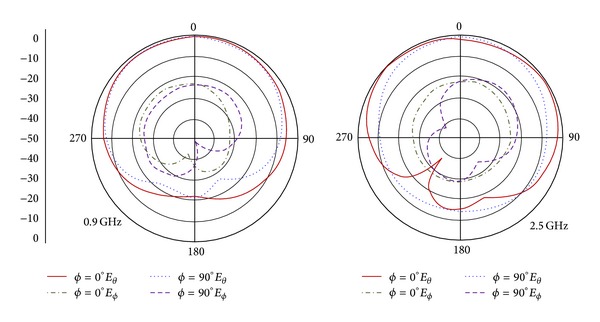
Radiation pattern for the proposed antenna for 0.9 GHz and 2.5 GHz.

Further study in terms of radiation characteristics has also been performed.  Figure 7 exhibits the far-field radiation patterns in *ϕ* = 0° and 90° for the frequencies at 0.9 GHz and 2.5 GHz, respectively. In the typical case of monopole antenna, the radiation pattern in the *ϕ* = 0° plane is marginally widespread than that of the *ϕ* = 90° plane which is readily observable from the figure. For lower frequency, the copolarization (*E*_*θ*_) patterns for both planes are almost symmetrical and directional. However, in the event of higher frequency, the effects of copolarization (*E*_*θ*_) and cross-polarization (*E*_*ϕ*_) over the off-boresight angles for *ϕ* = 0° are much disturbed due to the fact of abnormal current phase along the length of the antenna. However, the effect of cross-polarization for both *ϕ* = 0° and 90° plane at high frequency is increased to some extent since the variation of the current phase suppresses the excitation responsible to increase cross-polarization effect. Typically, excitation from higher order modes distorts the electric currents more specifically near to feed-radiating element joint which leads to degraded radiation. Use of meander-microstrip-feed essentially diminishes this issue as seen in the current distribution patterns in Figure 6. Through critical analysis, it can be conclude that the designed meandered-microstrip-fed vertical slot-loaded circular monopole antenna performs well by providing a nearly conformal radiation pattern radially for operating bands by maintaining low cross-polarization.

**Figure 8:**
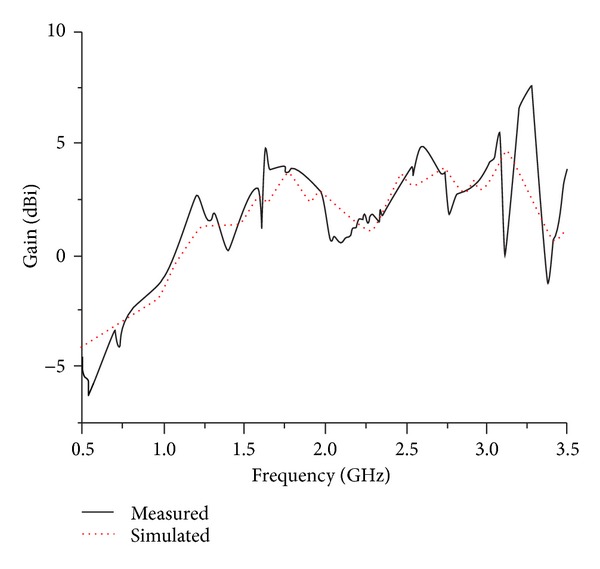
Simulated and measured gain of the proposed antenna.

**Figure 9:**
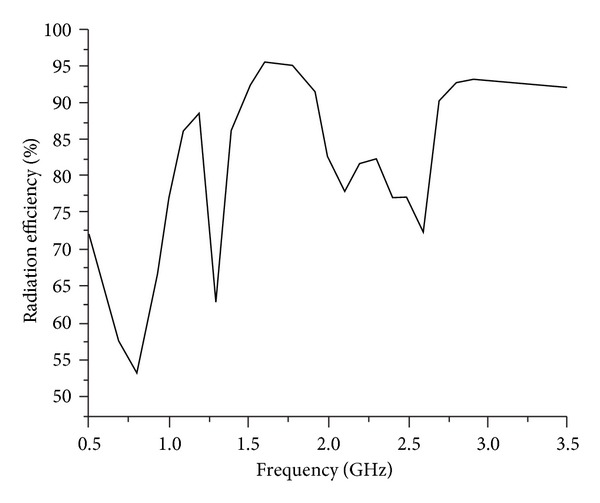
Simulated radiation efficiency of the proposed antenna.

Free-space ranges are used to measure the gain of the designed dual band antenna by utilizing two identical horn antennas whose gain and radiation patterns are known.  Figure 8 shows the measured and simulated gains against the corresponding operating frequency bands. For the lower operating band at 0.6 GHz–1 GHz, the average and maximum gains are −3.34 dBi and −1.18 dBi, respectively, whereas for the upper band at 2.25–2.95 GHz, the average and maximum gains are 3.15 dBi, 4.87 dBi, and 2.85 dBi, respectively, and this is why directivity of the designed antenna increased at high frequency.  Figure 9 exhibits the simulated radiation efficiency of the proposed antenna. It has been observed that maximum radiation efficiency 76.6% is achieved at a lower UHF RFID band with average efficiency over the band being 63.0%. On the contrary, for WiMAX/WLAN band the achieved average and maximum radiation efficiencies are 82.0% and 92.6%, respectively.

A comparative study has been performed between the proposed antenna and some existing antennas on the basis of dual band monopole characteristics, presented in Table 1. The tabulated data clearly show that the proposed antenna is comparatively smaller in dimension with enhanced bandwidth and relatively higher gain with nearly steady pattern performance. On the contrary, some of the specified antennas have reported a higher gain with compromised bandwidth and dimension. Additionally, these antennas would require more space to accommodate inside, small, portable device which is not a preferable situation.

## 5. Conclusion

A new slot line loaded round radiating monopole antenna with meandered-microstrip feed structure has been proposed in this paper for UHF RFID and WiMAX/WLAN bands application. The design parameters for the antenna have been critically analysed through EM simulator to achieve the optimum geometric structure for the prototype antenna. The experimental results represent the enhanced bandwidth that have been achieved for using the partial ground assisted meandered-microstrip fed structure; 44.4% for the lower band and 28% for the upper band with respect to their corresponding lower frequency cut at −10 dB. Concerning the antenna coverage and communication quality in terms of radiation and gain, the proposed antenna exhibits excellences with good dual band property which may help to find its prospective application in UHF-RFID and WiMAX/WLAN applications.

## Figures and Tables

**Table 1:** Comparison between the proposed antenna and some existing monopole dual band antennas.

Reference	Overall dimension, (mm^3^)	Center frequency, (GHz)	Bandwidth, (MHz)	Fract. bandwidth (%)	Gain (dBi)	Relative dielectric const., *ε*_*r*_	Application
[7]	50 × 100 × 5	0.718, 1.9	50, 155	6.26, 8.16	−2.4, −0.64	4.55	RFID
[8]	50 × 30 × 1.6	2.45, 5.5	124, 1124	5.1, 22.4	1.9, 4.3	4.4	WLAN
[31]	25 × 50 × 1.6	2, 2.45	270, 140	13.6, 5.7	1.8, 1.3	4.4	PCS/WLAN
[12]	108 × 108 × 1.6	0.87, 2.45	220, 600	26.2, 22.2	3.5, 4.2	4.4	LTE/PCS
Proposed	40 × 50 × 2	0.9, 2.5	400, 700	44.4, 28	−1.18, 4.87	14	RFID/WLAN
